# Mokko Lactone Attenuates Doxorubicin-Induced Hepatotoxicity in Rats: Emphasis on Sirt-1/FOXO1/NF-κB Axis

**DOI:** 10.3390/nu13114142

**Published:** 2021-11-19

**Authors:** Alaa Sirwi, Rasheed A. Shaik, Abdulmohsin J. Alamoudi, Basma G. Eid, Ahmed K. Kammoun, Sabrin R. M. Ibrahim, Gamal A. Mohamed, Hossam M. Abdallah, Ashraf B. Abdel-Naim

**Affiliations:** 1 Department of Natural Products, Faculty of Pharmacy, King Abdulaziz University, Jeddah 21589, Saudi Arabia; asirwi@kau.edu.sa (A.S.); gahussein@kau.edu.sa (G.A.M.); hmafifi@kau.edu.sa (H.M.A.); 2 Department of Pharmacology and Toxicology, Faculty of Pharmacy, King Abdulaziz University, Jeddah 21589, Saudi Arabia; rashaikh1@kau.edu.sa (R.A.S.); ajmalamoudi@kau.edu.sa (A.J.A.); beid@kau.edu.sa (B.G.E.); 3 Department of Pharmaceutical Chemistry, Faculty of Pharmacy, King Abdulaziz University, Jeddah 21589, Saudi Arabia; akammoun@kau.edu.sa; 4 Batterjee Medical College, Preparatory Year Program, Jeddah 21442, Saudi Arabia; sabrin.ibrahim@bmc.edu.sa; 5 Department of Pharmacognosy, Faculty of Pharmacy, Assiut University, Assiut 71526, Egypt; 6 Department of Pharmacognosy, Faculty of Pharmacy, Al-Azhar University, Assiut Branch, Assiut 71524, Egypt; 7 Department of Pharmacognosy, Faculty of Pharmacy, Cairo University, Cairo 11562, Egypt

**Keywords:** doxorubicin, mokko lactone, liver, Sirt-1

## Abstract

Doxorubicin (DOX), a common chemotherapeutic agent, suffers serious adverse effects including hepatotoxicity. Mokko lactone (ML) is a guainolide sesquiterpene with promising biological activities. The study aimed to evaluate the protection offered by ML against hepatotoxicity induced by DOX in rats. Our data indicated ML exhibited protective effects as evidenced by ameliorating the rise in serum activities of alanine transaminase, aspartate transaminase and alkaline phosphatase. This was confirmed histologically as ML prevented DOX-induced pathological alteration in liver architecture. Further, ML administration significantly prevented malondialdehyde accumulation, glutathione depletion and superoxide dismutase and catalase exhaustion. Antioxidant action of ML was associated with enhanced expression of the nuclear translocation of NF-E2-related factor 2 (Nrf2) and a lower expression of forkhead box protein O1 (FOXO1). Also, ML showed potent anti-inflammatory activities highlighted by decreased expression of interleukin 6, tumor necrosis factor α and nuclear factor κB (NF-κB). The anti-apoptotic effects of ML were associated with decreased Bax and enhanced Bcl-2 mRNA expression in liver tissues. ML caused a significant up-regulation in the expression of silent information regulator 1 (Sirt-1). Therefore, it can be concluded that ML prevents liver injury caused by DOX. This could partially be due to the ML regulatory activities on Sirt-1/FOXO1/NF-κB axis.

## 1. Introduction

Doxorubicin (DOX) is a broad-spectrum anthracycline antibiotic commonly utilized in combination with several drugs for treating different tumors, including solid tumors, lymphomas, and leukemias [1]. However, DOX clinical utilization is relatively restricted because of its serious toxicity to various organs such as the liver, heart, kidney, lung, testis and nervous system [2,3,4]. DOX is metabolized in the liver by cytoplasmic reductase and cytochrome enzymes to doxorubicinol and other toxic metabolites that can cause liver injury [5]. Several reports indicate that DOX treatment is associated with histopathological changes affecting liver tissues such as hepatocyte vacuolation, focal necrosis, cellular edema, bile duct hyperplasia and lymphocyte infiltration [6]. DOX therapy is also associated with increased serum levels of alkaline phosphatase (ALP), aspartate transaminase (AST) and alanine transaminase (ALT) indicating liver damage and necrosis [7]. The release of reactive oxygen species (ROS) during DOX metabolism is a culprit behind its hepatotoxicity. These ROS lead to exhaustion of antioxidant enzymes resulting in redox imbalance and oxidative stress [8]. Another culprit is apoptosis induced by DOX via increasing apoptotic protein expression [9]. Inflammation is also triggered by DOX as indicated by the elevated expression of several proinflammatory cytokines in liver tissues [10]. Therefore, prohibition of oxidative stress, apoptosis and inflammation could be an effective strategy for preventing DOX hepatotoxicity.

Intensive research efforts are carried out to explore the potential of natural compounds in disease treatment and prevention. Natural products constituted 16% of total drug approvals by the US-FDA in 2018, highlighting research efforts in this field [11]. Sesquiterpene lactones constitute a group of plant secondary metabolites which could be further subdivided into structurally distinct groups including guaianolides [12]. Detected in a spectrum of plants, guaianolides represent a variety of biologically active sesquiterpene lactones that can serve as defense molecules [13]. It has been also shown that guaianolides have many therapeutic effects such as anti-inflammatory, antixodiant, and antimicrobial activities [14]. The ability of guainolides to affect cell signaling may be due to the α-methylene-γ-lactone group, as it reacts with cysteine sulfhydryl groups of cellular peptides and proteins [15]. In this regard, *Costus speciosus* rhizomes are an excellent source of guaianolides sesquiterpenes. The rhizomes and roots are ascribed in Indian traditional medicine as anthelmintic, anti-inflammatory, antidiabetic, hepatoprotective, antihyperlipidemic, antispasmodic, and antimicrobial activities. The hepatoprotection of the plant has been referred to its constituents of guaianolides sesquiterpenes like costunolide and mokko lactone (ML) [16]. ML (dihydrodehydrocostus lactone) is utilized in several ailments including jaundice [17,18]. Also, ML exhibits potent anti-inflammatory activity as evidenced by significant reduction of IL-6 and TNF-α release from stimulated human peripheral blood mononuclear cells [19].

Because of the great importance of Dox in cancer chemotherapy, several approaches have been pursued to attenuate its toxicity. These included dosage optimization and use of analogs or combined therapy. However, no satisfactory outcomes have been obtained yet [20]. Thus, natural products are considered as an attractive approach to tackle to this problem [21]. Therefore, the current study aimed to investigate the potential protective activity of ML, purified from *Costus speciosus* rhizomes extract, against DOX-induced hepatotoxicity in rats.

## 2. Materials and Methods

### 2.1. General Experimental Procedures

ESIMS spectrum was measured utilizing a LCQ DECA mass spectrometer. BRUKER AVANCE 600 was utilized for NMR spectra measuring. Chromatographic separation was performed on SiO_2_ 60 (0.04–0.063 mm) and RP-18 (0.04–0.063 mm). TLC analysis was performed on pre-coated TLC plates with SiO_2_ 60 F_254_ (0.2 mm). The compound was detected by UV absorption (λ*_max_*255 and 366 nm), followed by spraying with *p*-anisaldehyde:H_2_SO_4_ and heating at 110 °C.

### 2.2. Plant Material

In March 2020, *C. speciosus* rhizomes were purchased from an authorized local market in Jeddah governorate, KSA. The plant’s authentication was proved by Dr. Emad Al-Sharif, (King Abdulaziz University, Faculty of Science & Arts) and a voucher specimen (no. CS-2-2020) was kept in the Faculty of Pharmacy’s herbarium.

### 2.3. Extraction and Isolation

**Figure 1:**
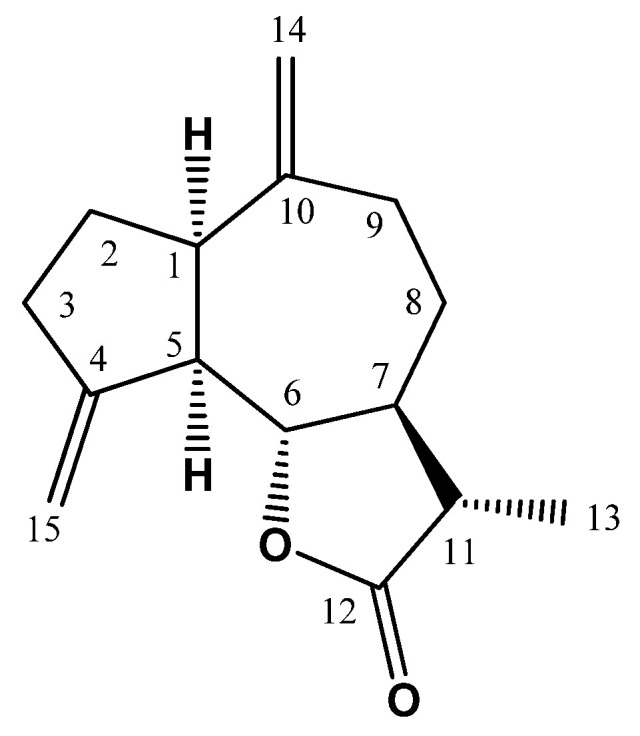
Chemical structure of ML.

The dried rhizomes (7.0 kg) were grinded and extracted with CHCl_3_ (6 times, 20 L each). The CHCl_3_ extract was separated from the marc using sterile gauze (cotton) and then filtered through No. 1 Whatman filter paper. Under reduced pressure, the solvent was removed to yield a viscous brown residue (445 g) that was suspended in 200 mL distilled H_2_O. Then, it was successively partitioned between *n*-hexane (8 × 2.0 L each) and chloroform (2.0 L × 8). Each fraction was concentrated to yield *n*-hexane (113.0 g) and chloroform (302.0 g) fractions. The *n*-hexane fraction was separated on SiO_2_ column utilizing gradient EtOAc:*n*-hexane to afford ML, that was further purified using RP-18 column (H_2_O:MeOH gradient) to produce ML as a yellow crystals (Figure 1).

### 2.4. Spectral Data of ML

Yellow crystals; ESIMS *m*/*z*: 233 [M+H]^+^; ^1^H NMR (CDCl_3_, 600 MHz): δ_H_ 5.20 (H-15A), 5.05 (H-15B), 4.88 (H-14A), 4.78 (H-14B), 3.92 (H-6), 2.88 (H-1), 2.81 (H-5), 2.54 (H-3, 8), 2.48 (H-9A), 2.11 (H-9B), 2.21 (H-11), 1.97 (H-7), 1.92 (H-2A), 1.83 (H-2B), 1.23 (H-13); ^13^C NMR (CDCl_3_, 150 MHz): δ_C_ 178.7 (C-12), 151.2 (C-4, 10), 107.8 (C-15), 111.9 (C-14), 85.3 (C-6), 52.0 (C-5), 49.9 (7), 47.1 (C-1), 42.1 (C-11), 37.7 (C-9), 32.5 (C-3, 8), 30.2 (C-2), 13.2 (C-13). All data are available in the Supplementary Materials (Figures S1–S3).

### 2.5. Chemicals

DOX was obtained from Sigma-Aldrich (St. Louis, MO, USA). Remaining chemicals had highest chemical grade.

### 2.6. Cell Viability Assay

MTT assay was performed to assess cell viability in two cell lines, HepG2 and HEK293. Both cell lines were maintained on EMEM supplement with 10% fetal bovine serum at 37 °C with 5% CO_2_ and split when 80% cellular confluency was reached. HepG2 and HEK293 cells were seeded in 96-well plates at a density of 2 × 10^3^ cell per well and incubated overnight. ML was dissolved in dimethyl sulfoxide (DMSO) then diluted in the cell culture medium at different concentrations. Then, it was added to the cells and incubated for 24, 48, and 72 h. At the end of incubation, the culture medium was aspirated and replaced with MTT solution at a final concentration of 0.2 mg/mL in each well, and the incubation was continued for 3 h at 37 °C. After that, the MTT solution was removed, and 100 μL DMSO solvent was added to dissolve the formazan crystals and incubated for 5 min. The absorbance was read at a 570 nm wavelength with a microplate reader. The percent cell viability relative to the vehicle control was calculated using the following equation:

% Cell Viability = (Absorbance of treated cells/Absorbance of control untreated cells) × 100.

### 2.7. Animals

Twenty-four male Wistar rats (200–230 g) utilized in this investigation were purchased from the animal facility, Faculty of Pharmacy, King Abdulaziz University. Animals were kept at 12-h light/dark cycle at (22 ± 3 °C) and humidity (≈60%) with free access to water and food. Experimental and animal-care protocols were approved by Research Ethics Committee, Faculty of Pharmacy, King Abdulaziz University (Approval Reference # PH-1443-13).

### 2.8. Experimental Design

Rodents were divided randomly into four groups (n = 6): control group, DOX group, and one treatment group received 15 mg/kg ML plus DOX and the other treatment group received 30 mg/kg ML plus Dox. ML doses were based on a pilot study and rat oral doses of the structurally related compound costunalide [22]. Briefly, the control group and DOX group consecutively received an oral dose of 0.5% carboxymethyl cellulose (CMC) once daily for 10 days. Treatment groups received ML suspended in CMC orally at the assigned doses for 10 days. 60 min after ML administration on the tenth day, the control group received an intraperitoneal (IP) injection of 0.9% saline, while other groups received DOX dissolved in 0.9% saline (15 mg/kg) via intraperitoneal injection. Dosing volumes in all animals were 10 mL/kg. Twenty four hours after the last dose, rodents were anesthetized using IP ketamine (50 mg/kg) and xylazine (5 mg/kg). Samples of blood were drawn from the retro-orbital plexus, left for 15 min and centrifuged at 3000 RPM for 10 min at 4 °C to separate sera. Animals were then sacrificed by decapitation and dissection of the liver was performed, which was then rinsed gently with ice-cooled saline. Then it was blotted between filter papers. Sections of the liver were kept in 10% neutral formalin for immunohistochemistry and histopathology. Another part of the livers was kept in RNAProtect Tissue Reagent (Cat. No. 76106, Qiagen, Germantown, MD, USA). Liquid nitrogen was used to flash-freeze the remaining sections and stored together with sera at −80 °C for subsequent examination.

### 2.9. Assessment of Hepatic Function Serum Markers

Serum alanine aminotransferase (ALT), aspartate aminotransferase (AST) and alkaline phosphatase (ALP) activities in the serum were assessed by colorimetric kits (AS 1031, AS 1061 and AP 1020 respectively, Biodiagnostic, Giza, Egypt).

### 2.10. Histopathological Examination

Tissues from the liver were placed in 10% neutral formalin for fixation and then embedded in paraffin. Subsequently the tissues were sectioned into slices (5 µm) and stained using hematoxylin and eosin (H&E). A light microscope (Nikon Eclipse TE2000-U, Nikon, Japan) was then used to photograph the sections. This examination was conducted by an expert pathologist without prior knowledge of the assigned groups.

### 2.11. Assessment of Oxidative Status

Liver tissues were homogenized in a ten-fold volume of ice-cooled phosphate-buffered saline (PBS, 50 mM potassium phosphate, pH 7.4). The homogenates were then centrifuged for 15 min at 10,000 and 4 °C. This was followed by the collection of the supernatant which was used for oxidative stress analysis. Commercially available kits were utilized to assess liver content of malondialdehyde (MDA, Cat. No. MD 2529, Biodiagnostic, Giza, Egypt) and reduced glutathione (GSH, Cat. No. GR 2511, Biodiagnostic, Giza, Egypt), and enzyme activities of superoxide dismutase (SOD, Cat. No. SD 2521, Biodiagnostic, Giza, Egypt) and catalase (CAT, Cat. No. CA 2517, Biodiagnostic, Giza, Egypt).

### 2.12. Immunohistochemical Staining

After deparaffinization, serial dilutions of ethanol were used to rehydrate tissue sections before boiling them for 10 min in 0.1 M citrate buffer (pH 6.0). Next the sections were kept in 5% bovine serum albumin (BSA) in tris buffered saline (TBS) for two hours. Tissue sections were then incubated with the primary antibodies to TNFα (Cat. No.: ab220210, Abcam^®^, Cambridge, UK), IL-6, (Cat. No.: ab9324, Abcam^®^, Cambridge, UK), NFκB (Cat. No.: sc-8414, Santa Cruz, TX, USA), Nrf2 (Cat. No.: MBS9608128, MyBioSource, San Diego, CA, USA), and FOXO1 (Cat. No.: sc-374427, Santa Cruz, TX, USA) at 4 °C for 12 h. Following flushing with TBS, the tissue sections were incubated with either anti-mouse or anti-rabbit biotinylated secondary antibody according to the primary antibody reactivity (Cell & Tissue Staining Kit, Cat. No.: CTS002, CTS006, R&D systems, Minneapolis, MN, USA). Image quantification was done using image analysis software (Image J, 1.46a, NIH, Bethesda, MD, USA) with a minimum of three sections per rat.

### 2.13. Quantitative Real-Time Polymerase Chain Reaction (PCR) Assay

RNA from liver tissues was isolated using TRIzol. RNA purity was assessed according to A260/A280 ratio, whereas RNA samples with ratios of more than 1.7 were included in cDNA synthesis. First-strand cDNA was synthesized using Omniscript RT kit (Cat. No.: 205113, Qiagen, MD, USA) before being subjected to qPCR to quantify changes in mRNA using a SYBR Green Master Mix (Cat. No.: 180830, Qiagen, MD, USA) containing the respective forward and reverse primers. Forward primers for Bax, Bcl-2, and β-actin were 5′CCTGAGCTGACCTTGGAGCA, 5′TGATAACCGGGAGATCGTGA, and 5′TCCGTCGCCGGTCCACACCC, respectively. Reverse primers for Bax, Bcl-2 and actin are 5′GGTGGTTGCCCTTTTCTACT, 5′AAAGCACATCCAATAAAAAGC, and 5′TCACCAACTGGGACGATATG, respectively. Data were normalized to β-actin, analyzed using the ΔΔCT method [23].

### 2.14. Western Blotting Assay

Protein lysate was prepared from liver tissues incubated for 30 min in ice-cold RIPA buffer supplemented with protease and phosphatase inhibitor cocktails before centrifugation at 4000 rpm for 20 min at 4 °C. After protein quantification with Protein Assay Kit I (Cat. No.: 5000006, Bio-Rad, Hercules, CA, USA), protein lysates (50 µg) were separated by a 10% Tris-Glycine gel and then placed on a PVDF membrane (Cat. No.: ab133411, Abcam, Cambridge, UK). using a semidry transfer cell (Bio-Rad) for 2 h. After blocking with 5% nonfat dry milk in TBST (10 mM Tris (pH 7), 100 mM NaCl, 0.1% Tween 20), membranes were kept overnight at 4 °C with Anti-Sirt-1 antibody (Cat. No.: ab110304, Abcam, Cambridge, UK). Membranes were thoroughly washed and incubated with rabbit anti-mouse HRP-conjugated secondary antibody at 1/10,000 (Cat. No.: ab6728, Abcam, Cambridge, UK) for one hour at room temperature. Immunoreactivity was detected using Enhanced Chemiluminescence kit on X-ray film (GE Healthcare, Piscataway, NJ, USA). The expression levels of Sirt-1 were normalized to β-actin detected using an anti-β-actin antibody (Cat. No.: ab8226, Abcam, Cambridge, UK) and the rabbit anti-mouse HRP-conjugated secondary antibody.

### 2.15. Statistical Analysis

All results are expressed as means ± SD. Results were assessed by one-way ANOVA followed by Tukey’s multiple comparison test (Prism 8.1, GraphPad Software, Inc., La Jolla, CA, USA). *p* < 0.05 was considered significant.

## 3. Results

### 3.1. Assessment of Cytotoxicity

**Figure 2:**
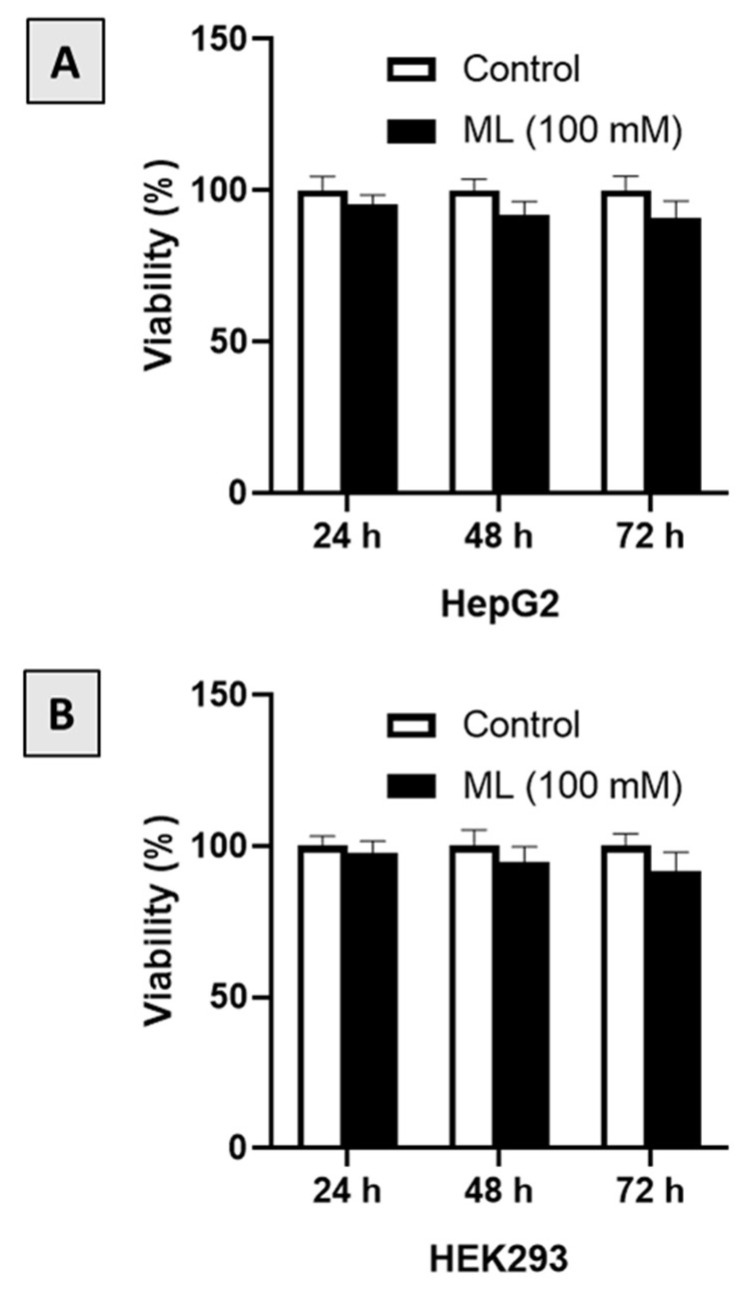
Cytotoxic effects of ML on HepG2 (**A**) and HEK293 (**B**) cells after exposure for 24, 48 and 72 h as determined by MTT assay. Data presented as mean ± SD.

Preliminary screening of ML against the cancerous cell line (HepG2) and the non-cancerous cells (HEK293) indicated that ML is non-cytotoxic. Therefore, the effect of a relatively high concentration of ML (100 μM) was assessed at 24, 48 and 72 h after starting incubation. As can be seen from Figure 2A, ML showed no significant cytotoxicity to HepG2 cells at all-time points. Similar findings were observed with HEK293 cells, where the viability of these cells did not change significantly following exposure to ML (Figure 2B).

### 3.2. Assessment of Liver Function

**Figure 3:**
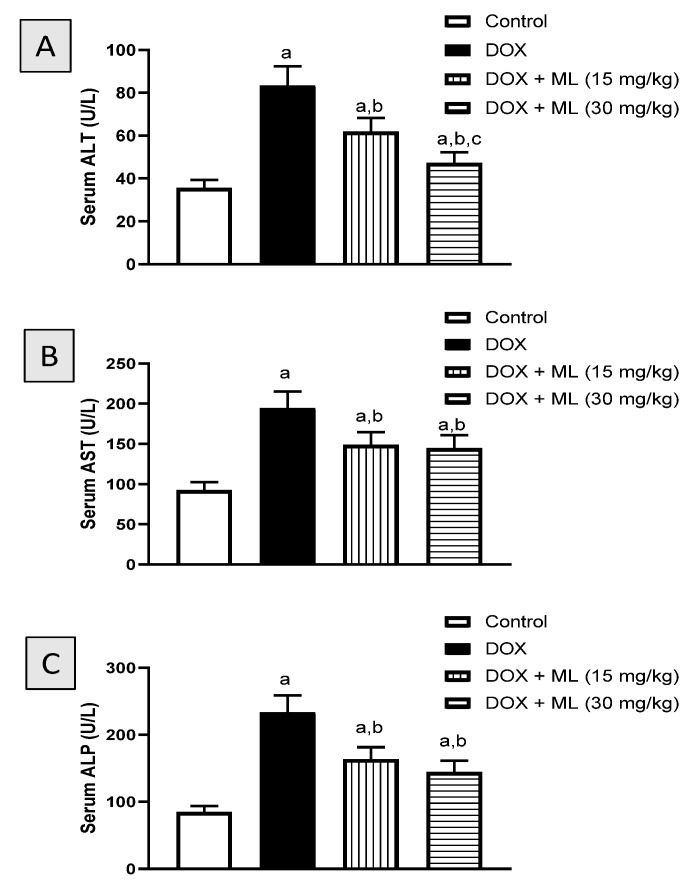
Effect of ML on serum markers of hepatotoxicity induced by DOX in rats. (**A**): Serum ALT activity, (**B**): Serum AST activity, (**C**): Serum ALP activity. Data are presented as Mean ± SD (n = 6). a: Significantly different from Control at *p* < 0.05; b: Significantly different from DOX at *p* < 0.05; c: Significantly different from DOX + ML (15 mg/kg) at *p* < 0.05.

The first set of experiments investigated the protective effects of ML (Figure 1) at two different doses on the hepatotoxicity induced by DOX in rats. Figure 3A shows that administration of DOX significantly increased serum ALT activity (134.6%). However, ML significantly ameliorated the increase in ALT activity by 25.8% and 43.4% at 15 mg/kg and 30 mg/kg, respectively. Treatment with DOX alone resulted in a 110.6% increase in serum AST levels compared to controls. Yet prior administration of ML at the two doses used inhibited the increase in serum AST activity associated with DOX by approximately 24% (Figure 3B). Serum ALP followed a similar pattern as presented in Figure 3C. Treatment with ML significantly reduced serum ALP activity induced by DOX by 29.9% and 38.0% at 15 mg/kg and 30 mg/kg, respectively.

### 3.3. Histopathological Examination

**Figure 4:**
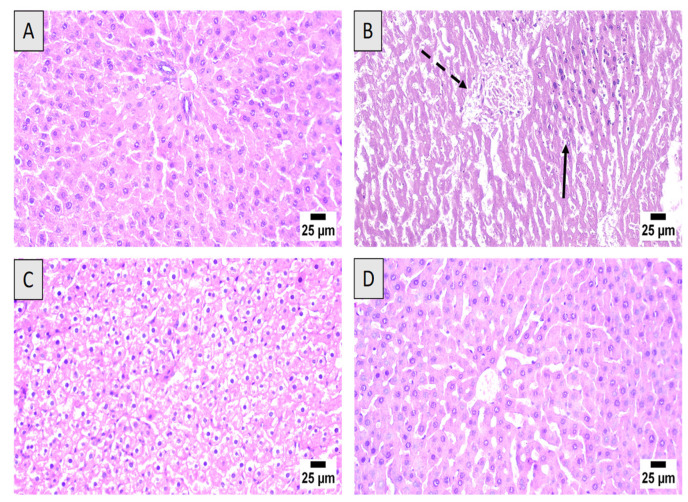
Histological examination of hematoxylin-eosin sections of rat livers. (**A**) Control group showing normal histoarchitecture of the liver tissues. (**B**) DOX-alone treated group with widespread hepatocellular necrosis (dashed arrow) and inflammatory cells infiltration (regular arrow). (**C**) DOX + ML (15 mg/kg) treated group exhibiting diffuse vacuolar degeneration of hepatocytes with limited necrotic areas. (**D**) DOX + ML (30 mg/kg) treated group showing apparently almost normal hepatic parenchyma.

Microscopically, liver tissues from control animals had normal histology, with hepatocytes having a normal lobular appearance with central veins surrounded by radiating hepatic cords. A normal histology was observed in the portal triads with hepatic artery branches, hepatic portal vein and bile duct (Figure 4A). Marked hepatotoxicity was observed in rats exposed to DOX-alone group. The hepatic parenchyma showed widespread hepatocellular necrosis associated with inflammatory cells infiltration and accumulation of eosinophilic and karyorrhectic debris (Figure 4B). Animals treated with ML (15 mg/kg) prior to DOX injection exhibited swollen hepatocytes with vacuolar degeneration and few individual cell necrosis (Figure 4C). The highest improvement of hepatic parenchyma was detected in DOX + ML (30 mg/kg) group. Several examined sections revealed apparently normal hepatic architecture, infiltration of scattered inflammatory cells without obvious microscopic alterations (Figure 4D).

### 3.4. Assessment of Oxidative Status

The next set of experiments was carried out to determine the protection offered by ML on oxidative status in DOX-induced hepatotoxicity in rats. Data in Table 1 indicate MDA, a product of polyunsaturated fatty acids peroxidation, was increased by DOX challenge 635.1% of the control value. On the other hand, ML ameliorated this increase associated with DOX by approximately one and two quarters at 15 mg/kg and 30 mg/kg, respectively. With regards to GSH content, the groups treated with ML and DOX had significantly higher GSH content in comparison to the group treated only with DOX. GSH content in the 15 mg/kg and 30 mg/kg ML groups was 47.2% and 72.3% higher than the DOX-only group, respectively. Hepatic SOD activities in groups received ML and DOX were significantly more than the levels found in the DOX group by 52.8% and 123.6% at ML doses of 15 mg/kg and 30 mg/kg, respectively. CAT activity had a similar pattern increasing by 82.9% and 94.7% when ML was administered prior to DOX at 15 mg/kg and 30 mg/kg, respectively.

### 3.5. Assessment of Nrf2 and FOXO1 Expression

**Figure 5:**
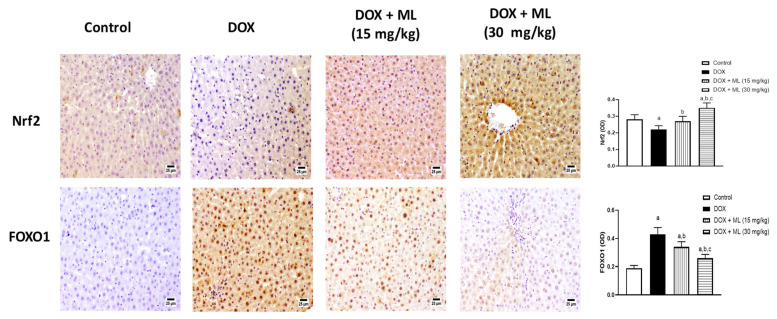
Effect of ML on expression of Nrf2 and FOXO1. Immunohistochemical photomicrographs of liver sections demonstrating the effect of ML on DOX-induced alteration in Nrf2 and FOXO1. Data presented in bar charts are Mean of optical densities ± SD (*n* = 6). a, b or c: Statistically different from Control, DOX or DOX + ML (15 mg/kg), respectively at *p* < 0.05.

The antioxidant activity of ML was confirmed by assessing the expression of Nrf2 and FOXO1 in hepatic tissues following ML treatment against DOX-induced hepatotoxicity. The top panel of Figure 5 shows that DOX challenge resulted in a significant decrease in Nrf2 expression. However, ML treatment at 15 mg/kg prevented the significant decreases in Nrf2 associated with DOX, whereas treatment with ML at 30 mg/kg markedly augmented the expression of Nrf2 compared to the control value. The bottom panel of Figure 5 shows the dose-related effects of ML on the expression status of FOXO1. Compared to the DOX-alone group, ML at 15 mg/kg caused a significant decrease in the DOX-induced expression of FOXO1 by 21.0%, while ML at 30 mg/kg has significantly reduced the expression of FOXO1 by 40.0%.

### 3.6. Assessment of Expression of Liver Inflammation Markers

**Figure 6:**
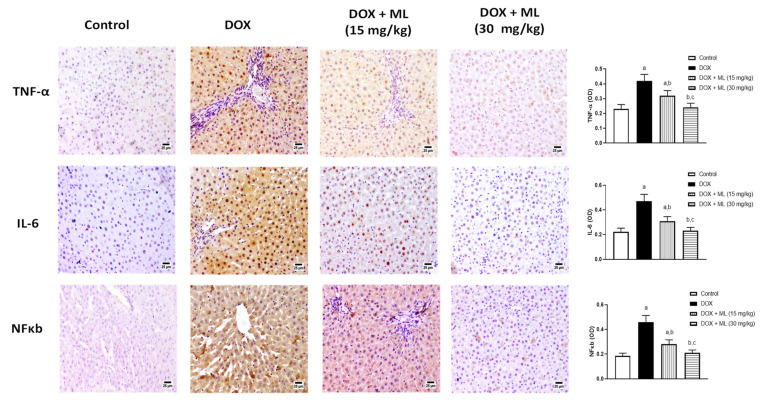
Immunohistochemical photomicrographs of liver sections demonstrating the effect of ML on DOX-induced alterations in IL-6, TNF-α and NFκB. Data presented in bar charts are Mean of optical densities ± SD (*n* = 6). a, b or c: Statistically different from Control, DOX or DOX + ML (15 mg/kg), respectively at *p* < 0.05.

The potential of ML as an anti-inflammatory agent was assessed in stressed liver tissues. The top panel in Figure 6 shows that co-administration of ML at 15 mg/kg significantly reduced the expression of TNF-α by 23.8% and at 30 mg/kg by 42.6% compared to DOX-alone group. IL-6 was greatly increased by the administration of DOX (middle panel of Figure 6). Yet, ML treatment significantly inhibited this increase by 34.0% and 50.6% at 15 mg/kg and 30 mg/kg, respectively. The bottom panel of Figure 6 shows that DOX challenge also caused a marked rise in NF-κB levels, and this increase was significantly attenuated by 39.1% and 54.3% by the prior administration of ML at 15 mg/kg and 30 mg/kg, respectively.

### 3.7. Assessment of mRNA Expression of Bax and Bcl-2

**Figure 7:**
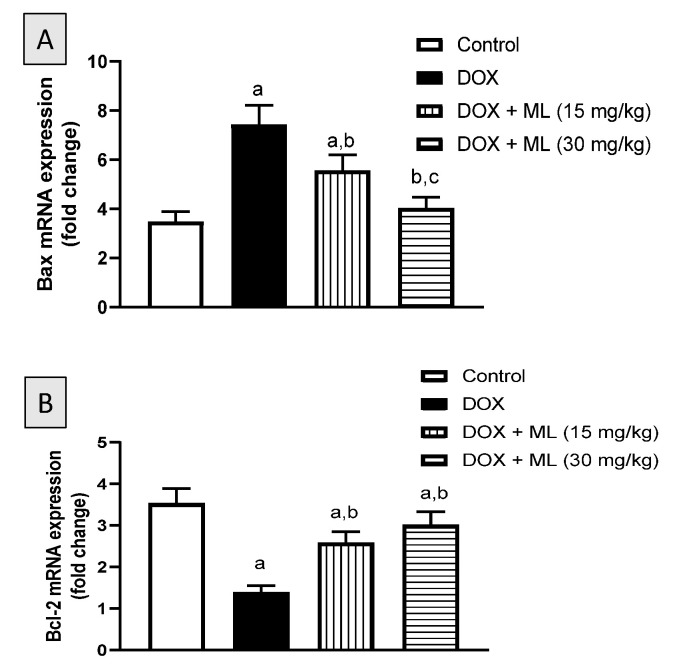
Effects of ML treatment on mRNA expression of Bax (Panel (**A**)) and Bcl-2 (Panel (**B**)) in the liver tissues. Data presented in bar charts are Mean of optical densities ± SD (*n* = 6). a, b or c: Statistically different from Control, DOX or DOX + ML (15 mg/kg), respectively at *p* < 0.05.

The anti-apoptotic activity of ML was explored by the assessing the mRNA expression of Bax and Bcl-2 in hepatic tissues challenged with DOX. As demonstrated in Figure 7A, the increase in mRNA expression of Bax associated with DOX treatment was significantly ameliorated by 25.0% and 45.6% following the co-administration of ML at 15 mg/kg and at 30 mg/kg, respectively. In contrast to the increase observed with Bax mRNA levels, treatment with DOX was associated with a 60.6% decrease in the mRNA expression of Bcl-2. Yet prior treatment with ML ameliorated this decrease in Bcl-2 mRNA expression associated with DOX by 55.9% and 70.6% at 15 mg/kg and 30 mg/kg, respectively (Figure 7B).

### 3.8. Evaluation of Sirt-1 Expression

**Figure 8:**
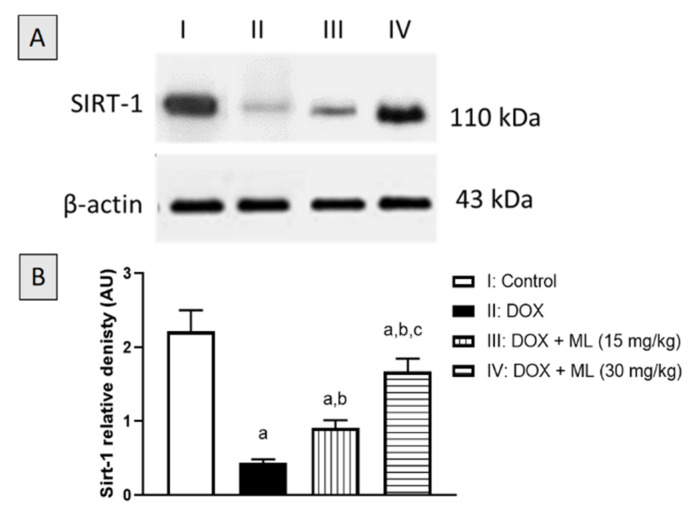
(**A**) Western blot analysis of Sirt-1 and β-actin expression in liver tissues. I: Control, II: DOX, III: DOX + ML (15 mg/kg); IV: DOX + ML (30 mg/kg). (**B**) Densitometric quantitation of Sirt-1. Data presented in bar charts are Mean of optical densities ± SD (*n* = 6). a, b or c: Statistically different from Control, DOX or DOX + ML (15 mg/kg), respectively at *p* < 0.05.

To gain further insight into the protective activity of ML in DOX-stressed liver tissues, the expression of the master regulator Sirt-1 was assessed after the DOX and ML treatment via Western blotting. As can be seen in Figure 8A,B, the protein expression of Sirt-1 was significantly decreased in DOX-treated livers by 80.5% compared to controls. On the other hand, ML treatment at the doses 15 mg/kg and 30 mg/kg significantly attenuated the decrease in Sirt-1 expression as it enhanced its values by 71.6% and 118.0% compared to DOX group, respectively.

## 4. Discussion

DOX is an established chemotherapy drug used in different cancers such as: lung, ovarian and breast cancers [24]. However, DOX is known to cause serious toxicities to non-tumor tissues including severe liver injury with jaundice [6]. ML is a naturally occurring sesquiterpene lactone purified from the rhizomes extract of *Costus speciosus*, a medicinal plant native to southeast Asia with many historical uses including fever, pneumonia and jaundice [17,18]. Therefore, this study set out with the aim of assessing the protection offered by ML against DOX-induced hepatic injury in rats. In vitro examination of ML in cancerous and non-cancerous cells indicated almost no cytotoxicity of the compound. On the experiments of serum markers of hepatotoxicity, our results indicated that co-administration of ML was able to ameliorate the elevated activities of ALT, AST and ALP at both doses tested relative to DOX-treated rats. In concordance with these reports, the histopathological examination of liver tissues from the groups that received ML prior to DOX was associated with relatively preserved hepatocytes, areas with mild lesions and comparable hepatic parenchyma morphology and inflammatory cell infiltration to the control group, indicating a protective activity of ML. These results are in line with many studies highlighting the hepatoprotective potential of sesquiterpene lactones. Several reports have shown that natural metabolites including terpenoids have the preventive capacity to limit DOX-induced liver damage. The sesquiterpene lactone artemisinin was reported to have protective activity against DOX-induced damage to heart and liver tissues in rats [25]. It was also shown that the fraction of *Taraxacum officinale* roots enriched in sesquiterpene lactones was effective in reducing oxidative stress and hepatotoxicity induced by carbon tetrachloride [26].

In the current investigation, DOX-induced liver injury was associated with oxidative stress. Yet it was found that ML co-administration significantly reduced DOX-induced oxidative stress as evidenced by reduced lipid peroxidation, GSH depletion and SOD and exhaustion of CAT. Our data gained support by the findings of a previous study showing that administration of the structurally related boswellic acids resulted in a significant improvement in oxidative stress markers and liver histopathological features in mice with DOX-induced liver damage [27]. The ability to restore intracellular redox balance of ML can be attributed to the reaction of its α-methylene-γ-lactone moiety with cysteine sulfhydryl groups of different cellular proteins [15]. These findings were further substantiated by showing that ML co-administration caused the expression of Nrf2 to increase significantly, which is a transcription factor with emerging roles in cell response to oxidative stress [28]. This is consistent with findings in the literature showing that Nrf2 is positively involved in regulating oxidative stress in DOX-induced cardiotoxicity [29,30]. These findings are suggestive of an Nrf2-dependent antioxidant response contributing to the hepatoprotective effects of ML in DOX-induced liver damage.

In addition, Nrf2 can directly increase resistance to apoptosis by inducing the expression of Bcl-2 [31]. ML in this study significantly ameliorated the decrease of Bcl-2 mRNA expression induced by DOX administration, hence this effect could be mediated by an Nrf2 dependent mechanism. Furthermore, ML co-administration has prevented the increase of the apoptotic Bax mRNA associated with DOX. Costunolide, a structurally related compound to ML, was also found to protect against apoptosis associated with oxidative stress via activating the Nrf2 signaling pathway [32,33]. These findings are consistent with those of another study demonstrating that costunolide alleviated hepatocytes apoptosis by upregulating Bcl-2 and downregulating Bax protein expression in a defense mechanism dependent on Nrf-2 expression [34]. Thus, the obtained data in this study suggest a central role of the antioxidant activity of ML in preventing apoptosis via the modulation of Nrf-2 and Bcl-2/Bax signaling axis.

The observed hepatoprotective effects in this study could be also mediated by the anti-inflammatory activity of ML as it was found to reduce TNF-α and IL-6 expression which key inflammatory mediators. In addition, ML significantly ameliorated the increase of NF-κB and FOXO1 expression associated with DOX-induced liver damage. The current findings concur with previous reports highlighting the potent anti-inflammatory activity ML as shown by the decreased release of IL-6 and TNF-α from human peripheral blood mononuclear cells [19]. It is known that FOXO1 increases the activation of NF-κB target genes such as TNF-α and IL-6 [10,35]. Hence, the anti-inflammatory effects associated with ML could stem from ameliorating the increase in FOXO1 expression induced by DOX which in turn ameliorated activation of NF-κB and the subsequent release of proinflammatory mediators. These results are consistent with the established anti-inflammatory effects of costunolide, as it halts NF-κB activation and proinflammatory cytokines expression [36,37,38].

Finally, our data show that ML co-administration mitigated the decrease in expression of Sirt-1 associated with DOX. Several reports have indicated the importance of Sirt-1-mediated regulation against hepatoxicity especially regarding oxidative stress, inflammation, and apoptosis [39]. It has been shown that Sirt-1 is involved in controlling hepatic inflammation via the regulation of FOXO1/NF-κB [10,40]. Sirt-1 is also known to be involved in the upregulation of antioxidant genes such as SOD via histone deacetylation [41]. With regards to apoptosis, it has been reported that Sirt-1 can regulate apoptosis via Bcl-2 and Bax, hence the differential changes induced by ML in the mRNA expression of Bcl-2 and Bax could be mediated by Sirt-1 [42]. Therefore, it can be concluded that ML protects against DOX-induced liver injury. This can be partially attributed to ML regulatory activities on Sirt-1/FOXO1/NF-κB axis. Further investigations are required to confirm ML safety and its ability to prevent DOX hepatotoxicity in the clinical setting.

## Figures and Tables

**Table 1:** Effect of ML on oxidative status in DOX-induced hepatotoxicity in rats.

	Control	DOX	DOX + ML (15 mg/kg)	DOX + ML (30 mg/kg)
MDA (nmol/mg protein)	0.37 ± 0.04	2.72 ^a^ ± 0.30	2.04 ^a,b^ ± 0.23	1.34 ^a,b,c^ ± 0.16
GSH (nmol/mg protein)	21.64 ± 0.24	8.63 ^a^ ± 0.96	12.70 ^a,b^ ± 1.36	14.87 ^a,b^ ± 1.52
SOD (U/mg protein)	3.36 ± 0.43	1.23 ^a^ ± 0.14	1.88 ^a,b^ ± 0.20	2.75 ^a,b,c^ ± 0.30
CAT (U/mg protein)	4.79 ± 0.50	1.87 ^a^ ± 0.20	3.42 ^a,b^ ± 0.36	3.64 ^a,b^ ± 0.39

Data are expressed as Mean ± S.D (*n* = 6). ^a^: Statistically significant from Control at *p* < 0.05. ^b^: Statistically significant from DOX at *p* < 0.05; ^c^: Statistically significant from DOX + ML (15 mg/kg) at *p* < 0.05.

## Data Availability

Data is contained within the article and the Supplementary Materials.

## Supplementary Materials

The following are available online at https://www.mdpi.com/article/10.3390/nu13114142/s1, Figure S1: ESIMS of mokko lactone, Figure S2: ^1^H NMR spectrum of mokko lactone in CDCl_3_ (600 MHz), Figure S3: ^13^C NMR spectrum of mokko lactone in CDCl_3_ (150 MHz).
