# Supplementary material for: Mokko Lactone Attenuates Doxorubicin-Induced Hepatotoxicity in Rats: Emphasis on Sirt-1/FOXO1/NF-κB Axis
Source: Nutrients. 2021 Nov 19;13(11):4142. doi: 10.3390/nu13114142 (PMC8621765; doi:10.3390/nu13114142)
Supplement: Supplementary file 1 [file nutrients-13-04142-s001.zip › nutrients-1452232-supplementary.pdf]

Mokko Lactone Attenuates Doxorubicin-Induced Hepatotoxicity in Rats: Emphasis on Sirt-1/FOXO1/NF-κB Axis

Alaa Sirwi [1], Rasheed A. Shaik [2], Abdulmohsin J. Alamoudi [2], Basma G. Eid [2], Ahmed K. Kammoun [3], Sabrin R. M. Ibrahim [4,5], Gamal A. Mohamed [1,6], Hossam M. Abdallah [1,7] and Ashraf B. Abdel-Naim [2,*]

[1] Department of Natural Products, Faculty of Pharmacy, King Abdulaziz University, Jeddah 21589, Saudi Arabia; asirwi@kau.edu.sa (A.S.); gahussein@kau.edu.sa (G.A.M.); hmafifi@kau.edu.sa (H.M.A.)
[2] Department of Pharmacology and Toxicology, Faculty of Pharmacy, King Abdulaziz University, Jeddah 21589, Saudi Arabia; rashaikh1@kau.edu.sa (R.A.S.); ajmalamoudi@kau.edu.sa (A.J.A.); beid@kau.edu.sa (B.G.E.)
[3] Department of Pharmaceutical Chemistry, Faculty of Pharmacy, King Abdulaziz University, Jeddah 21589, Saudi Arabia; akammoun@kau.edu.sa
[4] Batterjee Medical College, Preparatory Year Program, Jeddah 21442, Saudi Arabia; sabrin.ibrahim@bmc.edu.sa
[5] Department of Pharmacognosy, Faculty of Pharmacy, Assiut University, Assiut 71526, Egypt
[6] Department of Pharmacognosy, Faculty of Pharmacy, Al-Azhar University, Assiut Branch, Assiut 71524, Egypt
[7] Department of Pharmacognosy, Faculty of Pharmacy, Cairo University, Cairo 11562, Egypt
* Correspondence: aaabdulalrahman1@kau.edu.sa

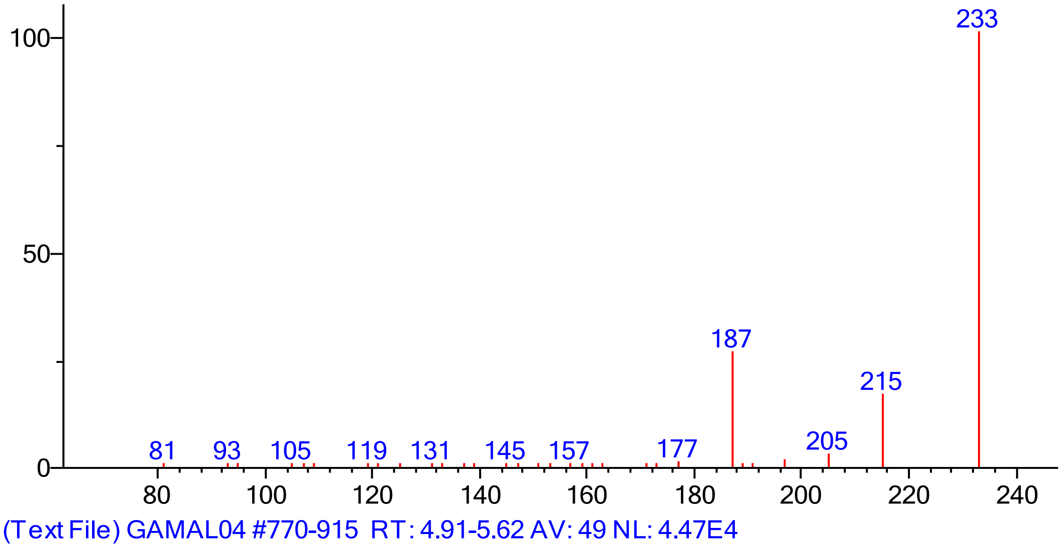

Figure S1. ESIMS of mokko lactone.

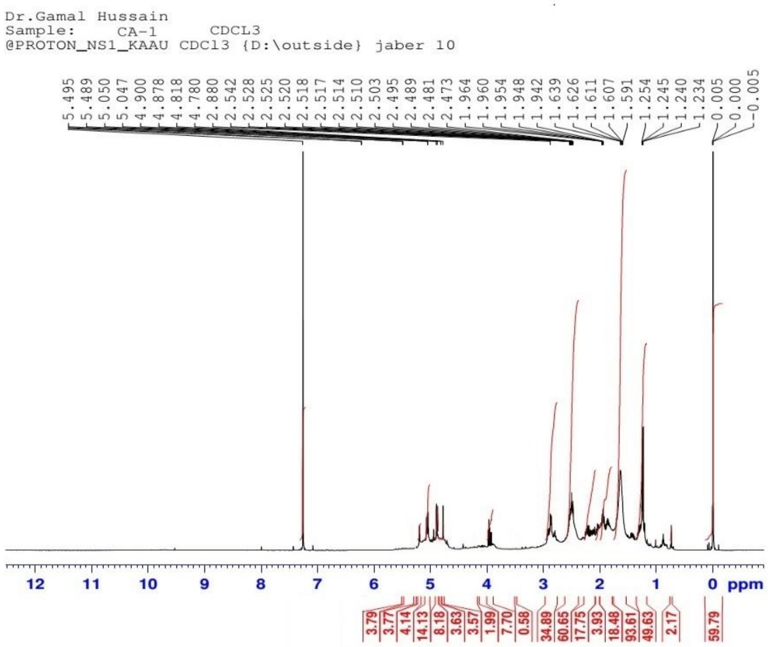

Figure S2. ^{1}H NMR spectrum of mokko lactone in $CDCl_3$ (600 MHz).

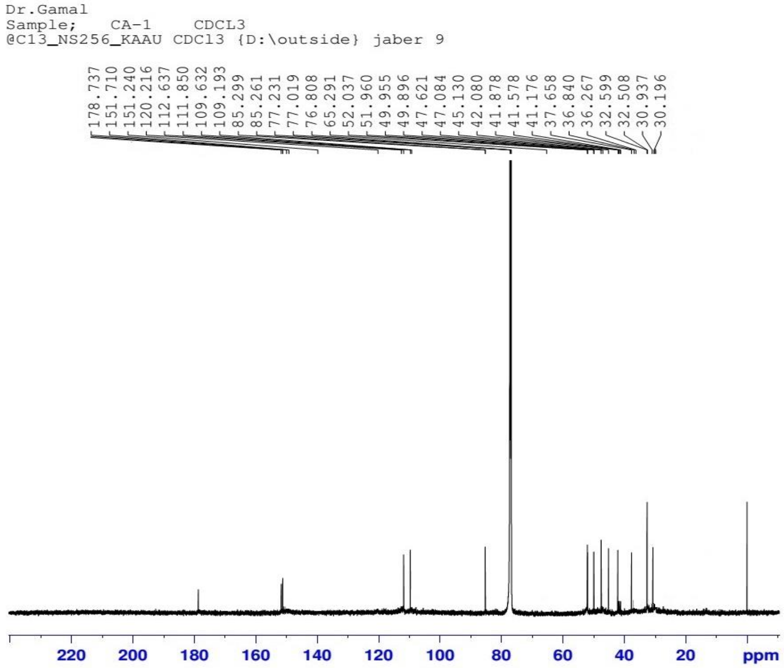

Figure S3. ^{13}C NMR spectrum of mokko lactone in $CDCl_3$ (150 MHz).
